# A Novel Biochar from Agro-Industrial Waste: Synthesis, Characterization, and Application for Acetylsalicylic Acid Removal

**DOI:** 10.1021/acsomega.5c08131

**Published:** 2025-11-18

**Authors:** Matheus Londero da Costa, Leandro Rodrigues Oviedo, Giovani Pavoski, Jorge Alberto Soares Tenório, Denise Crocce Romano Espinosa, Yolice Patricia Moreno, Daniel Moro Druzian, Sthéfany Nunes Loureiro, William Leonardo da Silva

**Affiliations:** † Applied Nanomaterials Research Group (GPNAp), 42510 Franciscan University (UFN), Santa Maria, RS 97010-032, Brazil; ‡ 28133 Polytechnical School of Chemical Engineering University of the Sao Paulo (USP), São Paulo, SP 05508-010, Brazil; § 28124 Strategic Technologies Center of Northeast (CETENE), Recife, PE 50740-545, Brazil; ∥ Department of Fundamental Chemistry (DQF), Federal University of Pernambuco (UFPE), Recife, PE 50670-901, Brazil

## Abstract

Due to drugs not being completely metabolized and excreted in their original form or as byproducts that contaminate water resources, the conventional treatment process often fails to remove these pollutants. Consequently, some studies seek to use waste to create materials capable of purifying water. Adsorption, being a process of low complexity and operational cost, stands out as a widely used process for wastewater treatment. In this context, the present work aims to synthesize and characterize a novel biochar from *Lagenaria siceraria* for the removal of the acetylsalicylic acid (ASA) drug by the adsorption process (kinetic, equilibrium, and thermodynamic) and to evaluate its ecotoxicity in *Artemia salina*. The biochar showed a heterogeneous, porous, and negatively charged surface (−18.76 ± 8.05 mV), with a surface area of 0.35 m^2^ g^–1^ and pH_ZCP_ = 6.95. It achieved 42% ASA removal under ideal conditions (10 mg L^–1^ ASA, 0.25 g L^–1^ biochar, and pH 4) and exhibited a maximum capacity (*q*_max_) of 137.33 mg g^–1^. In the thermodynamic study, there was a greater removal rate of 77% at 35 °C, and the best model was the Khan model in adsorption equilibrium (*R* ^2^ = 0.999), which indicates mono- and multilayer adsorption by electrostatic action. At low ASA concentrations, the pseudo-first-order (PFO) model presented the best fit, indicating a predominance of fast adsorption at surface sites and physical interactions. Meanwhile, for higher concentrations of ASA, the pseudo-second-order (PSO) model was more adequate, suggesting an increasing contribution of specific chemical interactions at higher-energy sites. The biosorbent showed no ecotoxicity between 100–1000 mg L^–1^ and a lethal dose of 100% ASA (L_D100_) at 300 mg L^–1^. Therefore, biochar can be used as an alternative material for the removal of organic pollutants, such as drug residues.

## Graphical abstract

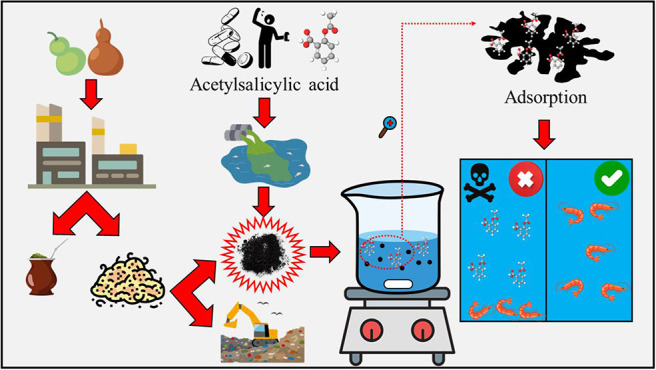

## 1 Introduction

Acetylsalicylic acid (ASA) is one of the most consumed drugs around the world (around 50 billion aspirin tablets are consumed each year worldwide),^1^ and it is sold in concentrations ranging from 75 to 500 mg L^–1^ around the world.^2,3^ As with any drug, ASA is not completely metabolized by the human body, achieving a metabolization rate of 80%, and the remaining amount is excreted unchanged or as byproducts, which in turn reach water bodies, contaminating the environment and affecting people.^4^

Moreover, ecotoxicity tests have been conducted to determine a lethal dose for simpler organisms,^5^ such as *Artemia salina* and *Daphnia magna*, which are crustaceans more sensitive to pollution and serve as a food base for other crustaceans and fish.^6,7^ Trials with *Daphnia magna* showed a lethal concentration (L_C_) of 88.33 mg L^–1^ of ASA after 48 h of exposure.^8^

In this view, there is a need for the development of advanced technologies for wastewater treatment, as conventional water treatment does not entirely remove pollutants due to the low biodegradation rate of 24–48 h.^9,10^ Thus, research has been carried out with Advanced Oxidative Processes (AOPs),^11^ membranes,^12^ and adsorption, which is the most commonly used method due to its low cost and complexity.^13^

In addition, for the adsorption process to occur in a favorable direction, it needs to have certain characteristics, such as being a nanometric material, as it will have a high surface area and a greater presence of pores, which will lead to a greater chance of contact between the adsorbent and the adsorbate, and a higher adsorption capacity.^14^ Thus, there are studies that verify the applicability of biochar originating from agro-industrial residues for the removal of drugs such as paracetamol,^15^ ibuprofen,^16^ sodium diclofenac,^17^ and dexamethasone.^18^

Agro-industrial waste is commonly generated during and after the harvesting process, which mainly consists of leaves, roots, bark, and pulp.^19^ Worldwide, the region that generates the most agro-industrial waste is found in Asia, with 47%, followed by the American continents with 29%, Europe with 16%, Africa with 6%, and Oceania with 2%.^20,21^

Moreover, agro-industrial waste, such as gourd, can be used as a promising adsorbent for the removal of pharmaceuticals from aqueous solutions, due to its ease of preparation and low cost.^22^ Then, the high-temperature carbonization (400–800 °C)^23^ commonly done in times of less than 2 h^24^ is a process in which a disruption of the original molecular structure of a given material is noticed when carbonaceous materials are subjected to high temperatures to obtain coal or activated carbon.^25^ Thus, carbonization is responsible for the opening of the porosity of the raw material, the removal of impurities (low-weight carbonaceous material is converted to CO_2_ and water steam), and the improvement of the chemical stability of the material.^26,27^

In this view, the present work aims to synthesize and characterize an adsorbent from agro-industrial residues from a gourd company by carbonization and a chemical activation process for application in the removal of the ASA drug. Moreover, this study carried out equilibrium, kinetic, and thermodynamic studies of adsorption and ecotoxicity assays using saline brine shrimp to evaluate the toxicity of the biochar and the lethal dose of the ASA. The novelty of the study lies in the use of waste from a gourd carving industry as a biosorbent to remove ASA effluent, thus promoting a circular economy, adding value to waste, and meeting the Sustainable Development Goals (SDGs). Furthermore, biochar emerges as a promising biosorbent, transforming organic waste into an eco-efficient solution with high added value for potential application in environmental remediation, redefining the treatment of wastewater with drugs.

## 2 Materials and Methods

### 2.1 Adsorbate Preparation

The solutions of the ASA commercial (Multilab) were prepared, ranging from 3.3–36.7 mg L^–1^, and were placed in the ultrasound (Unique-UltraSonic Cleaner model) for 30 min at 50 ± 2 °C.^28^

### 2.2 Synthesis of the Biochar

For the preparation of the biochar (Figure 1), a slow pyrolysis process was used with *Lagenaria siceraria* waste (local industry, Santa Maria-RS, Brazil). Thus, 30 g of in natura gourd (#20 Mesh Test Sieve – 850 μm) were mixed with 600 mL of 0.1 mol L^–1^ H_3_PO_4_ (Synth, ACS, 85%) (activating agent) under magnetic stirring (835 rpm/30 min/60 °C).^29^ The pH was adjusted (pH 5.5) with 1 mol L^–1^ NaOH (Sigma-Aldrich, PA). Finally, the material was calcined at 600 ± 2 °C with a heating rate of 30 °C min^–1^ for 2 h in a muffle furnace^30^ (model EDG, f3000, Brazil). The material was sieved (#100 Mesh Test Sieve – 150 μm).

**Figure 1:**
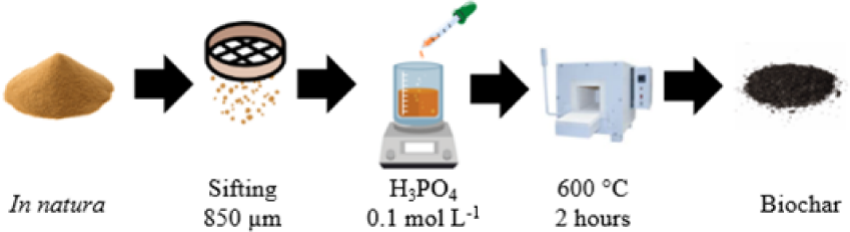
Schematic representation of the biochar synthesis process from *Lagenaria siceraria* waste.

### 2.3 Adsorption Experiments

100 mL of the ASA solution (3.3–36.7 mg L^–1^) were mixed with the biochar (0.08–0.92 g L^–1^) and pH ranging from 2 to 12, according to the experimental design. The removal of the ASA drug was evaluated by monitoring the process, where aliquots (2 mL) were collected at predetermined times (0, 5, 15, 30, 45, 60, 75, and 90 min). All aliquots collected were filtered (0.22 μm filter, Millex GP) and analyzed by UV–vis spectrophotometer (Shimadzu UV Mini 1240 UV–vis Spectrophotometer-W2B) at the characteristic wavelength of ASA (λ = 327 nm), according to Figure S1 (Supporting Information—SI). Thus, the absorbance was related to the concentration of the ASA through a calibration curve: Abs = 1.048 [ASA] (*R* ^2^ = 0.999; *N* = 7). All tests were performed in duplicate (error value lower than 5%).

The efficiency of the biochar for the ASA removal was evaluated through its adsorption capacity, *q_t_* (mg g^–1^), according to eq 1,

1 $$q_{t}=\frac{\left(C_{0}-C_{t}\right)\cdot m}{V}$$

Where *C*_0_ and *C*_*t*_ are the initial and final concentrations of ASA drug in solution (mg L^–1^), *V* (L) is the volume of the solution, and *m* (g) is the mass of the biochar.

### 2.4 Characterization Techniques

X-ray Diffraction (XRD) was used to check crystallinity in a D2 PHASER XRD diffractometer with a copper tube (λ_α‑Cu_ radiation = 0.15418 nm) and an angular range of 10°–70°. Fourier-Transform Infrared Spectroscopy (FTIR) was used to determine the functional groups in a Shimadzu (IR Prestige-21) FTIR Spectrometer (ranging from 3600 to 500 cm^–1^) with a resolution of 4 cm^–1^. For the Dynamic Light Scattering (DLS), a NanoBrook Omni (Brookhaven Instrument Corporation, New York, USA) was used with a red laser diode (35 mV, λ = 640 nm) at a detection angle of 90°. The textural properties were determined by N_2_ porosimetry using a Micromeritics Gemini VII 2375 Surface Area Analyzer, where the specific surface area (*S*_BET_) was determined by the Brunauer–Emmett–Teller equation (BET) method, and the pore diameter and pore volume were carried out by the Barrett–Joyner–Halenda (BJH) method. For the Zeta potential (ZP), a Zetasizer Nano ZS (ZEN3600, UK) with closed capillary cells (DTS 1060) (Malvern Instruments, UK) and a 4 mW He–Ne laser (633 nm) was used. Field Emission Gun Scanning Electron Microscopy (FEG-SEM) was used for morphological analysis with a TESCAN Mira3 XMH Scanning Electron Microscope, with an acceleration voltage of 4 kV and a working distance of 10 mm, with a magnification of 10 kx. The zero charge point (pH_ZCP_) was determined by the 11-point methodology.^31^

### 2.5 Experimental Design

The three main factors that influence the process were chosen, such as the concentration of the pollutant (ASA), the adsorbent (Biochar), and pH, based on literature^32,33^ were defined by the central composite rotational design (CCRD 2^3^) methodology to determine the ideal condition with 3 central points and 6 axial points by using the Gauss–Newton algorithm^34^ using the Statistic 10 software (StatSoft, USA). Table 1 shows the points obtained by CCRD 2^3^.

**Table 1:** Central and Axial Points Were Determined by CCRD.2^3^

	–1.67	–1	0	1	+1.67
[ASA] (mg L^–1^)	3.3	10	20	30	36.7
[Biochar] (g L^–1^)	0.08	0.25	0.50	0.75	0.92
pH	2	4	7	10	12

### 2.6 Equilibrium Adsorption Study

The adsorption equilibrium study relates the availability of active sites on the biosorbent surface with the number of ASA drug molecules, where there are models (isotherms) that describe the adsorption capacity. In this study, the Langmuir^35,36^ (eq 2), Freundlich^37–40^ (eq 3), Sips^41–43^ (eq 4), Toth^44,45^ (eq 5), and Khan^46^ (eq 6) models were used.

2 $$q_{e}=\frac{q_{max}\cdot K_{L}\cdot C_{e}}{1+K_{L}\cdot C_{e}}$$

3 $$q_{e}=k_{F}\cdot {C_{e}}^{1/n}$$

4 $$q_{e}=\frac{q_{S}\cdot a_{S}\cdot C_{e}^{1/S_{p}}}{1+a_{S}\cdot C_{e}^{1/S_{p}}}$$

5 $$q_{e}=q_{max}.\frac{K_{T}.C_{e}}{({\left(1+{\left(K_{T}.C_{e}\right)}^{tn}\right)}^{1/tn}}$$

6 $$q_{e}=\frac{q_{max}\cdot \alpha_{k}\cdot C_{e}}{1+{(K_{k}\cdot C_{e})}^{\alpha_{k}}}$$

Where: *q*_*e*_ is the amount of solute adsorbed per gram of adsorbent at equilibrium (mg g^–1^); *q*_*max*_ the maximum adsorption capacity (mg g^–1^); *K*_*L*_ is the adsorbent–adsorbate interaction constant (L mg^–1^); *C*_*e*_ is equilibrium concentration of solute in solution (mg L^–1^); *K*_*F*_ is the Freundlich adsorption constant (mg^1–(1/n)^ (g^–1^) L^1/n^); *n* is the constant related to surface heterogeneity; *q*_*S*_ the monolayer adsorption capacity (mg g^–1^); *a*_*S*_ the Sips isotherm model constant (L g^–1^); *S*_*P*_ the Sips isotherm exponent; *K*_*T*_ is Toth isotherm constant (L mg^–1^); *tn* is a constant dimensionless associated with the heterogeneity of the adsorbent surface; *K*_*k*_ the Khan isotherm model constant (L mg^–1^) and *α*_*k*_ is Khan isotherm model exponent.

### 2.7 Adsorption Kinetics

Kinetic studies on the adsorption of solutes from aqueous solutions are applied to determine how fast the process occurs or at what rate equilibrium is reached.^47^ Thus, the best conditions of biochar concentration and pH value were fixed, while varying the ASA concentration (5, 10, 30, 50 mg L^–1^). Moreover, pseudo first-order (PFO),^48^ pseudo second-order (PSO),^49,50^ and intraparticle diffusion-adsorption models (ID)^51^ were used, according to the eqs 7–9, respectively.

7 $$q_{t}=q_{1}\cdot \left(1-e^{-k_{1}t}\right)$$

8 $$q_{t}=\frac{t}{\left(\frac{1}{k_{2}.q_{2}^{2}}\right)+\left(\frac{t}{q_{2}}\right)}$$

9 $$q_{t}=k_{id}\cdot t^{0.5}+C$$

Where: *k*_1_ (min^–1^) is the rate constant of pseudo-first-order; *q*_1_ (mg g^–1^) is the value of adsorption capacity for PFO; *k*_2_ (g mg^–1^ min^–1^) is the rate constant of pseudo-second-order; *q*_2_(mg g^–1^) is the value of adsorption capacity for PSO; *k*_*id*_ is the intraparticle diffusion rate constant (mg g^–1^ min^–0.5^); and *C* reflects the effect of the boundary layer or surface adsorption (mg g^–1^).

### 2.8 Adsorption Thermodynamics

Thermodynamic parameters were determined by the changes in Gibbs free energy (Δ*G* ^0^, kJ mol^–1^), entropy (Δ*S* ^0^, kJ mol^–1^ K^–1^), and enthalpy (Δ*H* ^0^, kJ mol^–1^), which indicate whether the process is spontaneous, exothermic, or endothermic^52^ (eqs 10–12).

10 $$\Delta G^{0}=-R\cdot T\cdot \ln K_{c}$$

11 $$\ln K_{c}=\frac{\Delta S^{0}}{R}-\frac{\Delta H^{0}}{RT}$$

12 $$K_{c}=\frac{q_{e}}{C_{e}}$$

Where: *K* is the equilibrium constant (L g*K_c_* ^–1^); *q*_*e*_ is the amount of solute adsorbed per gram of adsorbent at equilibrium (mg g^–1^); *C*_*e*_ is the concentration in solution (mg L^–1^); *T* is absolute temperature (298.15, 308.15, and 318.5 K); and *R* is the universal gas constant (8.314 J mol^–1^ K^–1^).

### 2.9 Statistical Analysis

The kinetic and equilibrium parameters were determined by fitting the models to the data using nonlinear regression, using the Statistic 10 software (StatSoft, USA) with the Quasi-Newton method. The coefficient of determination (*R* ^2^), adjusted coefficient of determination (*R* ^2^_adj_), root-mean-square error (RMSE), the sum of squared errors (SSE), and average relative error (ARE) were used to evaluate the model’s fit quality, according to eqs 13–17):^53–56^

13 $$R^{2}=1-\frac{\overset{N}{\underset{i=1}{\sum }}{(q_{\exp}-q_{\mathrm{pred}})}^{2}}{\overset{N}{\underset{i=1}{\sum }}{(q_{\exp}-{\mathrm{q̂}}_{\mathrm{pred}})}^{2}}$$

14 $$R_{\mathrm{adj}}^{2}=1-\left[\frac{\left(1-R^{2}\right)\cdot \left(n-p\right)}{\left(n-p-1\right)}\right]$$

15 $$RMSE=\sqrt{\frac{\overset{N}{\underset{i=1}{\sum }}{(q_{\exp}-{\mathrm{q̂}}_{\mathrm{pred}})}^{2}}{n}}$$

16 $$SSE=\frac{1}{n}\cdot \overset{n}{\underset{1}{\sum }}{(q_{\exp}-q_{\mathrm{pred}})}^{2}$$

17 $$ARE=\frac{1}{n}\cdot \overset{n}{\underset{1}{\sum }}\frac{(q_{\exp}-q_{\mathrm{pred}})}{q_{\exp}}$$

Where: *q*_exp_ and *q̂*_pred_ are the experimental and the predicted adsorption capacity (mg g^–1^), respectively; *q̂*_pred_ is the predicted data associated with the response (absorbance); *n* is the number of data; *p* is the number of model parameters.

### 2.10 Ecotoxicity Tests

#### 2.10.1 Preparation of Artificial Saline Water

For the preparation of artificial saline water, 1 L of distilled water was mixed with 23 g of NaCl (Synth), 11 g of MgCl_2_·6H_2_O (Synth), 4 g of Na_2_SO_4_ (Nuclear), 1.3 g of CaCl_2_·2H_2_O (Nuclear), and 0.7 g of KCl (Synth). Moreover, the pH of the solution was adjusted to 9 with a solution of Na_2_CO_3_ (Synth),^57^ which was measured using a benchtop pH meter (Ohaus pH Meter Model ST3100-F).

#### 2.10.2 Cyst Eclosion and Incubation Procedure

For the hatching of the cysts, a 1 L beaker was used, to which 500 mL of artificial saline water was added. The beaker was protected with aluminum foil, leaving only a small opening on the upper surface, and illuminated by an 18 W lamp at a distance of about 30 cm. The water was aerated for 15 min and then added 10 mg of *Artemia salina* cysts where the aeration was maintained throughout the hatching process using a commercial aquarium pump for 48 h with a flow rate of 3.5 L min^–1^ in a controlled temperature environment of 28 ± 2 °C, a salinity of 32 μg mL^–1^ and a pH 9.^58^

#### 2.10.3 Acute Toxicity and Lethal Concentration

For the determination of acute toxicity and lethal concentration (L_C_), a negative control (NC) was prepared, consisting of artificial saline water without any extra substance, and the positive control (PC) consisted of copper sulfate solution (CuSO_4_, 0.0006 mg mL^–1^) and finally the biochar concentrations ranged from 100 to 1000 mg L^–1^ and the ASA concentrations ranged from 50 to 350 mg L^–1^.^59^

Thus, the assays were carried out in triplicate in test tubes containing 10 mL of the test or control solution with 10 nauplii at 28 ± 2 °C for 48 h with a 12 h light/12 h dark photoperiod. Afterward, the dead nauplii or any signs of abnormal behavior (e.g., difficulty in swimming^60^ were counted.

## 3 Results and Discussion

### 3.1 Characterization

Figure 2 shows the XRD diffractograms of the *in natura* gourd and biochar, where crystalline peaks were identified: at 16.19° (101), 22.42° (002), and 34.67° (040), indicating the presence of hemicellulose, lignin, and cellulose,^61^ which disappear or reduce in intensity with the pyrolysis process in the development of biochar. Moreover, due to the calcination process (600 °C), the reactivity of phosphorus increases, forming possible functional groups on the surface of the biochar and allowing the formation of crystalline phases.^62^ It is worth noting that this is important for the adsorption of the pollutant, as the crystalline phase can maintain an organized structure, while the amorphous phase promotes more empty spaces to accommodate the ASA molecules, allowing an increase in diffusive stages (extra- and intraparticle), as well as mass transfer phenomena.

**Figure 2:**
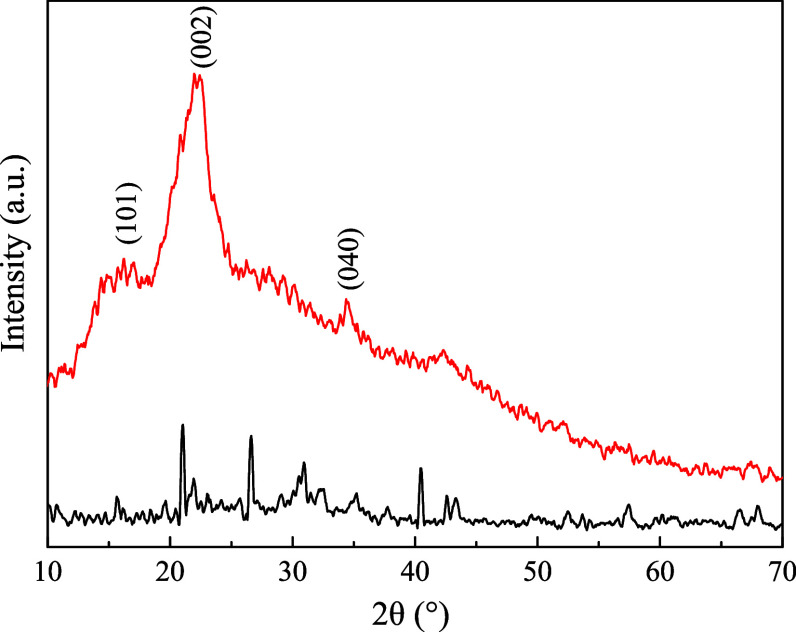
XRD pattern diffractograms of *in natura* gourd waste and biochar.

Figure 3 illustrates the FTIR spectrum of the biochar, where several key absorption bands can be identified:^63–69^ (a) at 3600 cm^– 1^ are assigned to the stretching vibration of O–H bonds relative to alcohols, carboxylic acids, phenols, or hydroxyl functional groups; (b) at 2500 cm^– 1^ and 1430 cm^– 1^ correspond to the C–H vibrational modes of methyl groups; (c) at 1800 cm^–1^ originate from either the C =O stretching vibration of carbonyl-related structures or the C–O vibration of carboxylic groups; (d) at 1685 cm^–1^ the axial stretching of – C= ; (e) C associated with −C–H (alkene and alkenyl side chains) in the region of 990 cm^–1^; and (f) the formation of an aromatic ring in the region of 750 cm^–1^.

**Figure 3:**
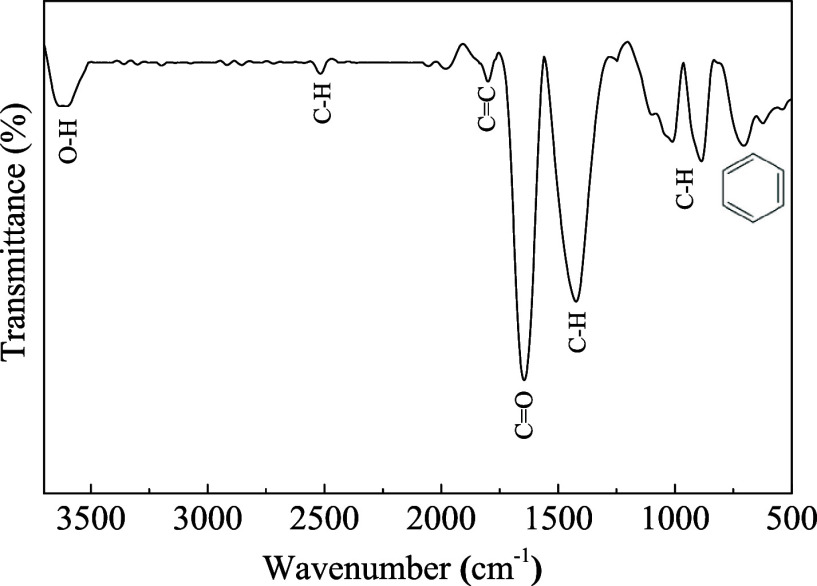
FTIR spectrum of biochar from *Lagenaria siceraria* waste.

Figures 4(a) and 4(b) represent the N_2_ adsorption/desorption isotherms of the *in natura* gourd and biochar, respectively. According to Figure 4(a), the biochar represents a type III isotherm, describing an adsorption process on macroporous adsorbents with strong and weak adsorbate-adsorbent interactions, respectively, while Figure 4(b) presents a type V isotherm with hysteresis type H3, which means that the material is mesoporous, with pores that are cracks between aggregated particles or lamellar structures.^70,71^

**Figure 4:**
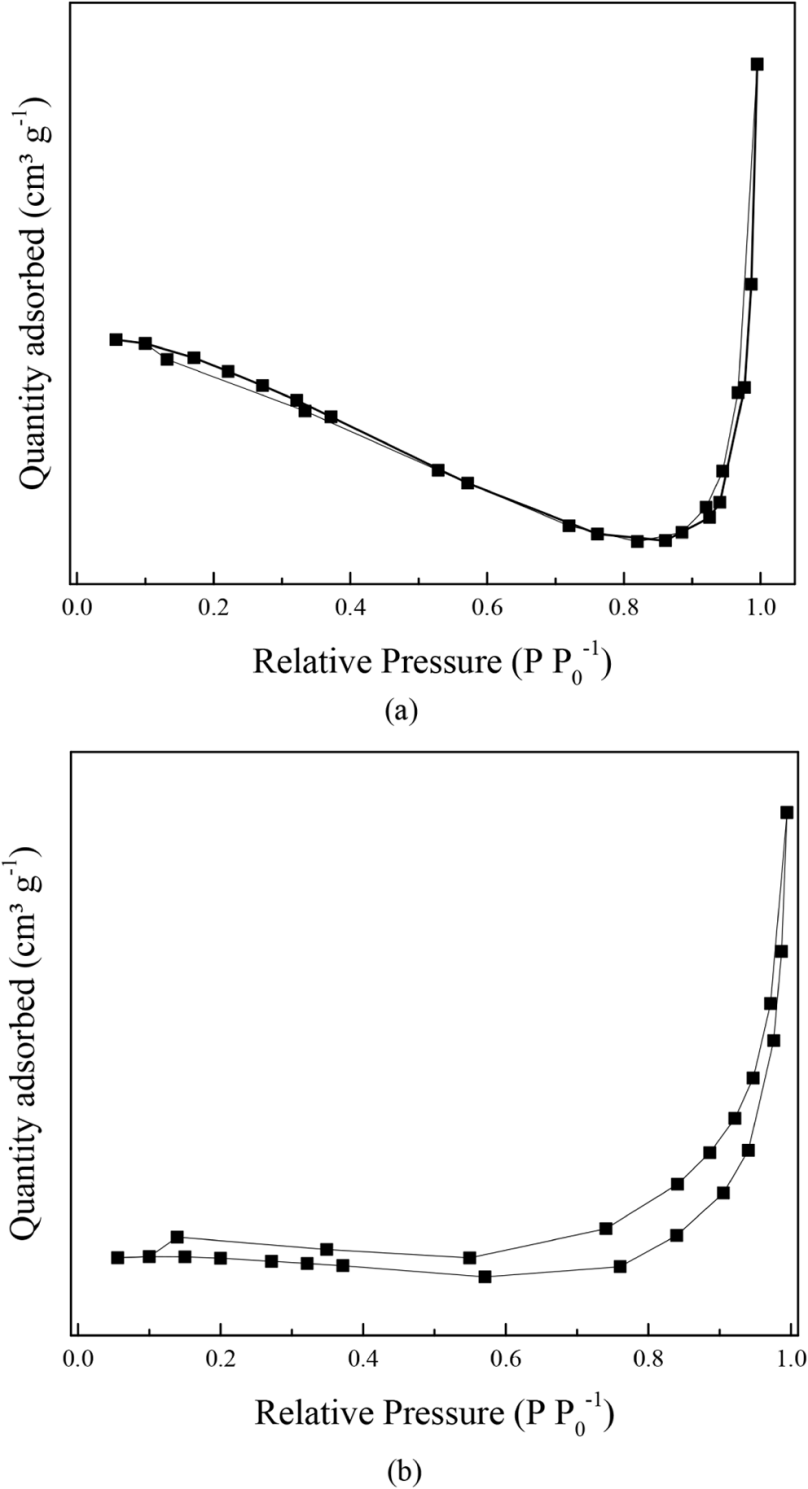
(a) N_2_ adsorption/desorption isotherms of (a) *in natura* gourd waste and (b) biochar.

In addition, the physical properties after the carbonization process of *in natura* material are shown in Table 2, indicating an increase in *S*_BET_ and *V*_p_ due to the removal of inorganic compounds, which were initially added to prevent the expansion or shrinkage of carbon molecules in the adsorbent during the carbonization and activation process. Moreover, the mesoporosity of materials can be confirmed by the values of the average pore diameter of these materials.^72,73^ Regarding ZP, all materials showed a negative charge due to the presence of carboxylic groups originating from hemicellulose, lignin, and cellulose in the *in natura* gourd waste.^74^ According to the DLS results (Figure 5), the average size of the agglomerated particles showed a significant reduction (from 1379.8 to 66.2 nm), indicating the colloidal stability of the biochar due to the thermal process (calcination) during its synthesis, which contributes to the adsorption process by providing greater availability of contact area between the biosorbent surface and the ASA drug.

**Table 2:** Specific Surface Area (*S*_BET_), Pore Diameter (*D*_p_), Pore Volume (*V*_p_), and Zeta Potential (ZP) of the *In Natura* Gourd Waste and Biochar

	*S*_BET_ (m^2^ g^–1^)	*D*_p_ (nm)	*V*_p_ (cm^3^ g^–1^)	ZP (mV)
*In natura*	0.09 ± 0.04	78.3 ± 2.07	0.002 ± 0.01	-19.96 ± 0.52
Biochar	0.35 ± 0.01	36.3 ± 9.77	0.002 ± 0.01	-18.76 ± 8.05

**Figure 5:**
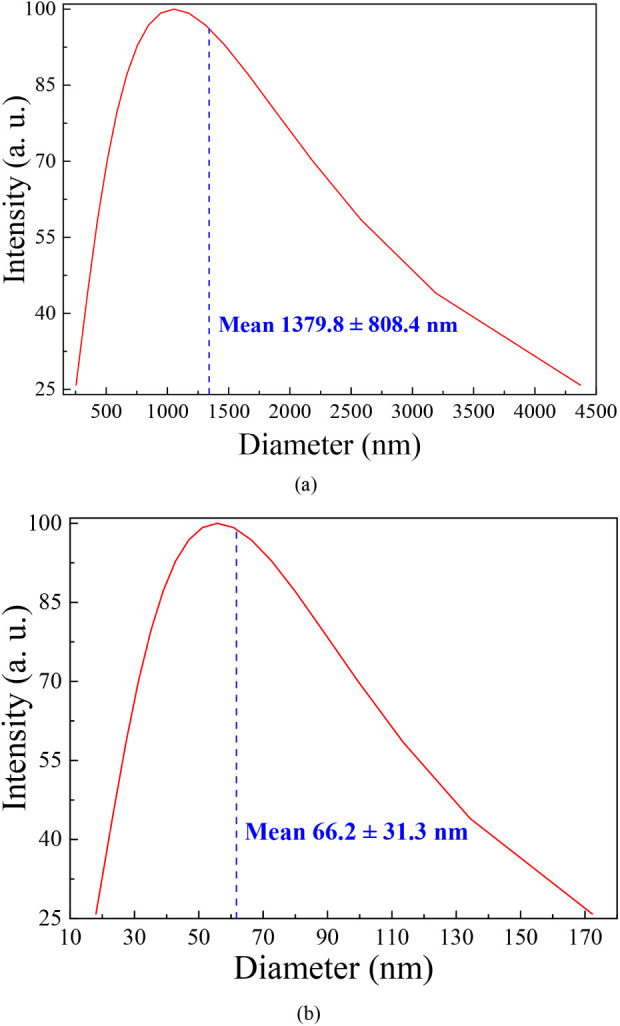
Distribution of particle size obtained from the DLS analysis for (a) *in natura* gourd waste and (b) biochar.

Figure 6 (a) and (b) presents the morphology of the materials through FEG-SEM micrographs of the *in natura* gourd waste and biochar, respectively. Figure 6(a) shows the morphology of the *in natura* with a disorganized structure without visible pores that contains fibers typically found in biomasses, while for biochar (Figure 6(b) showed a porous network structure is shown due to the activation and carbonization process, being an extremely suitable morphology for dye adsorption as it promotes the access of the ASA molecules to the internal structure of the adsorbent.^75^

**Figure 6:**
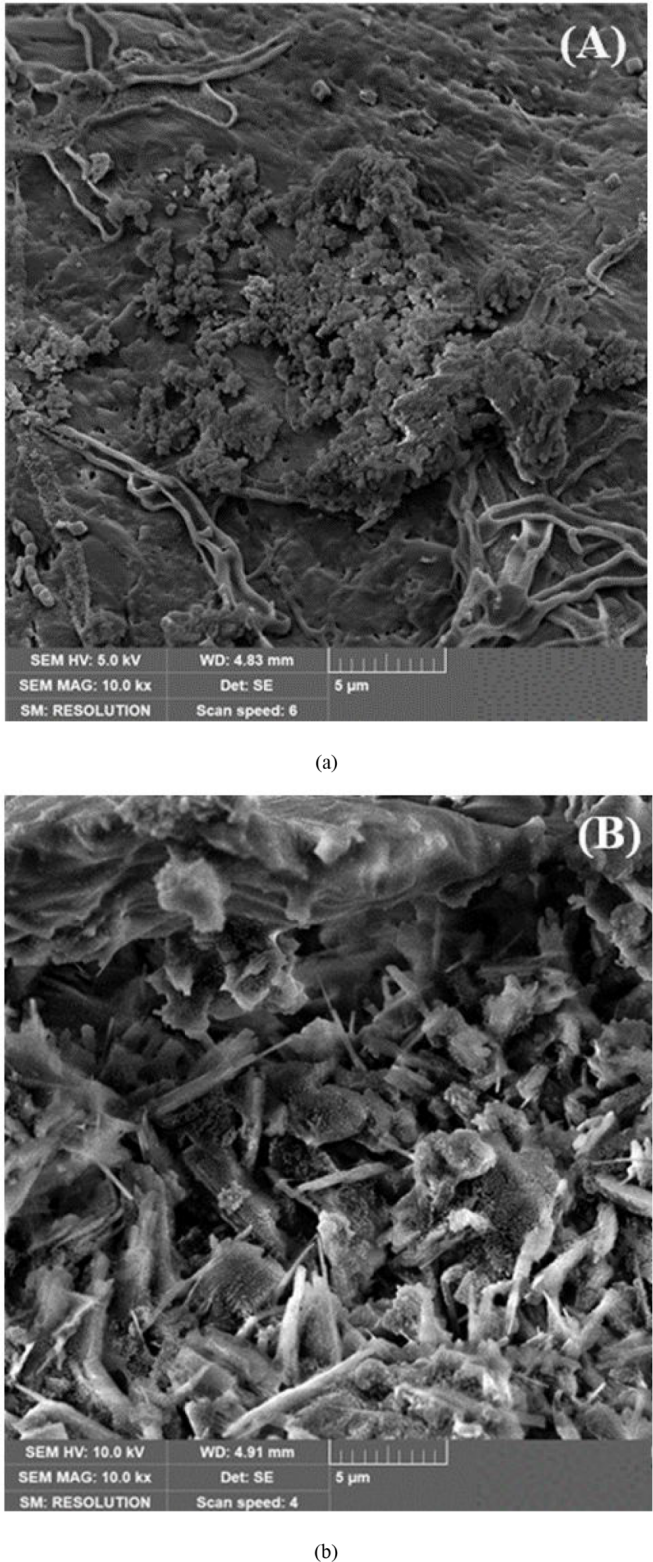
FEG-SEM micrographs with 10 kx magnification for (a) *in natura* gourd waste and (b) biochar.

Figure 7 shows the pH_ZCP_ of the biochar, which was 6.95, as found in the literature.^76^ When the pH of the solution is lower than this pH_ZCP_, it indicates that the surface of the biochar was protonated, thus aiding in the adsorption of negatively charged molecules (presence of carboxylic groups), and many anions were adsorbed to balance the positive charges. Otherwise, the adsorption process can be explained by the electrostatic attraction between the charge present in the adsorbent and the anionic group in the solution.^77^

**Figure 7:**
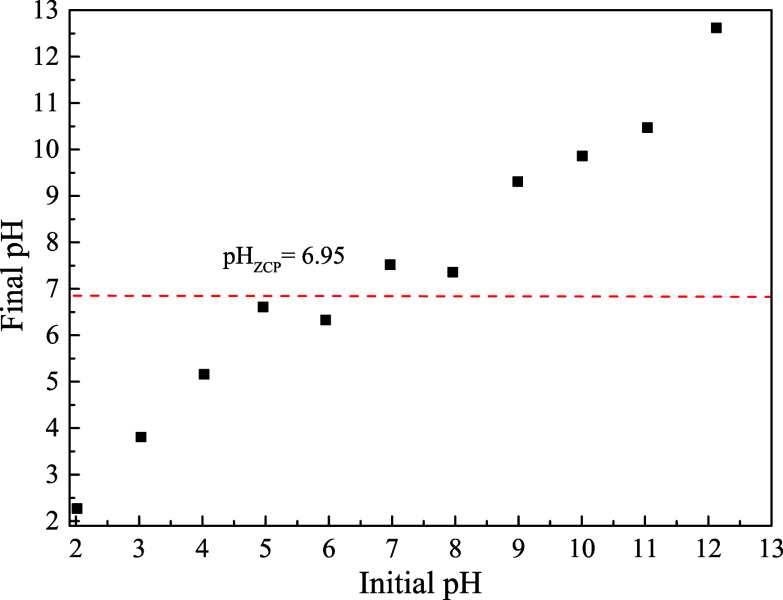
Zero charge point (pH_ZCP_) of the biochar from the *Lagenaria siceraria* waste.

### 3.2 Experimental Design

Figure 8 shows the Pareto chart for ASA removal, indicating that the independent variable affecting the adsorption process for removing the ASA drug was the concentration of biochar. Thus, the linear interaction of biochar concentration had a negative direct effect due to the electrostatic interactions and the selective interactions of the active sites available with the ASA molecules. Therefore, the ideal conditions for ASA removal by adsorption were 10 mg L^–1^ of ASA, 0.25 g L^–1^ of biochar, and pH 4.

**Figure 8:**
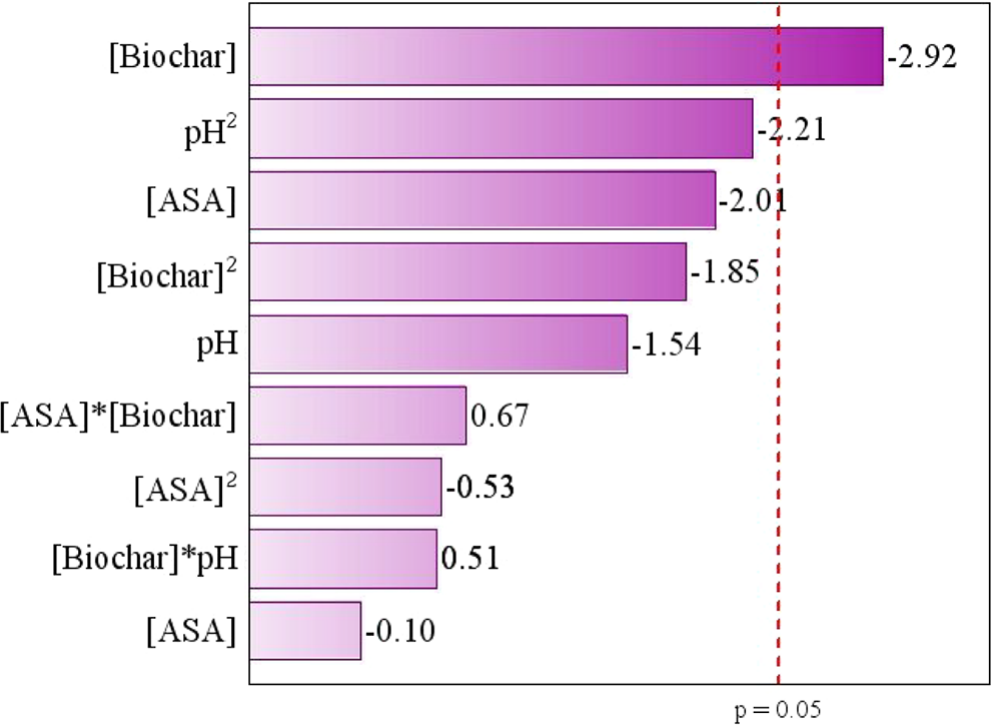
Pareto chart from CCRD 2^3^ for the removal of the ASA drug.

Table 3 shows the variance analyses (ANOVA) of the experimental design for ASA removal, where the main parameters and regression coefficients are denoted, resulting in a predicted model with an *F*_cal_ of 325 and an *F*_tab_ of 3.68, indicating 76.13% of the adjusted experimental data. The *R* ^2^ was 0.7613, possibly due to the material tending to agglomerate, which does not make the linear model predictable and can generate some prediction errors, thus decreasing the R^2^ value.

**Table 3:** Sum of Squares (SS), Degrees of Freedom (df), Mean Square Error (MS), *F*_cal_ (calculated Degrees of Freedom), *F*_tab_ (degrees of Freedom Tabulated), and Determination Coefficient (*R* ^2^) for ASA Adsorption

	SS	df	MS	*F*_cal_	*F*_tab_	*R* ^2^
[ASA]	707.844	1	707.844	325	3.68	0.7613
[ASA]^2^	51.153	1	51.153			
[biochar]	1487.127	1	1487.127			
[biochar]^2^	600.278	1	600.278			
pH	413.267	1	413.267			
pH^2^	858.754	1	858.754			
[ASA] * [biochar]	78.438	1	78.438			
[ASA] * pH	1.892	1	1.892			
[biochar] * pH	45.840	1	45.840			
Error	1222.786	7	174.684			
Total SS	5122.47409	16	–			

### 3.3 Adsorption Isotherms

The experimental data were fitted with Langmuir, Freundlich, Khan, Sips, and Toth adsorption isotherm models, according to Figure 9. The corresponding model parameters are listed in Table 4.

**Figure 9:**
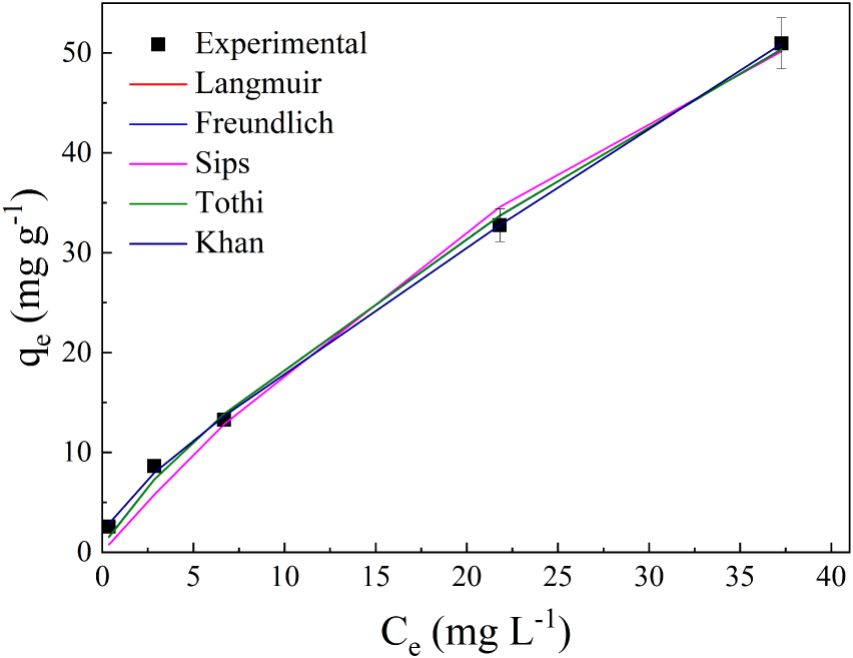
Equilibrium adsorption curves for adsorption of ASA drug onto biochar (biochar concentration = 0.25 g L^–1^ and pH 4).

**Table 4:** Equilibrium Parameters for the Adsorption of ASA Drug onto Biochar

**Langmuir**						
Parameter	Value	*R* ^2^	*R* ^2^_adj_	SSE	ARE (%)	RMSE
*q*_max_ (mg g^–1^)	137.33 ± 6.87	0.990	0.981	3.114	22.771	1.764
*K*_L_ (L mg^–1^)	0.0154 ± 0.001					

**Freundlich**						
*K*_F_ (mg^1–(1/*^n^*^) (g^–1^) L1/^*n*^)	3.33 ± 0.17	0.997	0.995	0.875	12.639	0.936
*n*	1.33 ± 0.07					

**Khan**						
*q*_max_ (mg g^–1^)	3.09 ± 0.15	0.999	0.999	0.136	4.562	0.369
*K*_K_	40.88 ± 2.04					
*a*_K_	0.34 ± 0.01					

**Sips**						
*q*_s_ (mg g^– 1^)	137.35 ± 6.87	0.990	0.981	3.114	22.713	1.764
*a*_S_ (L g^–1^)	1.016 ± 0.05					
*S*_p_	65.82 ± 3.29					

**Toth**						
*q*_max_ (mg g^– 1^)	0.015 ± 0.001	0.997	0.994	0.916	13.004	0.957
*K*_T_ (L mg^–1^)	9861.97 ± 493.1					
*t_n_*	0.26 ± 0.01					

According to Figure 9 and Table 4, the Khan model was the best fit for the experimental data, as shown in Figure 9 (*R* ^2^ and *R* ^2^_adj_ = 0.999), since this model generated lower error values (SSE, ARE, and RMSE) than the other isotherms tested. The Khan isotherm is particularly notable for its hybrid nature, incorporating characteristics of both the Langmuir and Freundlich models. It describes adsorption as occurring simultaneously in monolayer and multilayer forms. This implies that adsorption begins with a strong interaction at specific active sites (akin to the Langmuir model) but transitions to multilayer adsorption as the surface becomes saturated, driven by weaker adsorbate–adsorbate interactions (similar to the Freundlich model). This dual mechanism makes the Khan model especially suitable for systems where heterogeneous adsorption occurs due to a combination of surface site specificity and adsorbent interactions.^79^

### 3.4 Adsorption Kinetics

Table 5 shows the parameters evaluated from ASA adsorption kinetic studies, and Figure 10 shows the curve of each evaluated kinetic model. Thus, the ASA molecules’ adsorption onto the biochar surface occurred fast within the first 15 min, and the rate gradually decreased until equilibrium, due to the reduction of unoccupied adsorption sites and the decrease in the concentration gradient.

**Table 5:** Kinetic Parameters for the Adsorption of ASA Drug onto Biochar

Pseudo first-order model (PFO)							
[ASA] (mg L^**–**1^)	*q*_1_ (mg g^**–**1^)	*k*_1_ (min^**–**1^)	*R* ^2^	*R* ^2^_adj_	SSE	ARE (%)	RMSE
1	2.555 ± 0.128	3.321 ± 0.166	0.974	0.970	4 × 10^–17^	2 × 10^–8^	6 × 10^–9^
5	8.839 ± 0.442	0.360 ± 0.018	0.818	0.788	0.086	0.321	0.293
10	18.056 ± 0.903	0.022 ± 0.001	0.919	0.905	0.096	0.245	0.310
30	28.636 ± 1.432	18.213 ± 0.911	0.485	0.399	3 × 10^–22^	5 × 10^–12^	1 × 10^–11^
50	45.173 ± 2.259	19.924 ± 0.996	0.552	0.477	1 × 10^–18^	2 × 10^–10^	1 × 10^–9^

Pseudo second-order model (PSO)							
[ASA] (mg L^–1^)	*q*_2_ (mg g^–1^)	*k*_2_ (g (mg min)^**–**1^)	*R* ^2^	*R* ^2^_adj_	SSE	ARE (%)	RMSE
1	2.555 ± 0.128	9 × 10^–4^ ± 0.001	0.974	0.970	0.013	0.434	0.117
5	8.881 ± 0.444	0.187 ± 0.009	0.779	0.766	0.085	0.320	0.291
10	26.413 ± 1.321	6 × 10^–4^ ± 0.001	0.916	0.902	0.335	0.592	0.578
30	37.314 ± 1.866	3 × 10^–3^ ± 0.001	0.783	0.747	0.389	0.209	0.624
50	58.764 ± 2.938	2 × 10^–3^ ± 0.001	0.864	0.841	1.089	0.222	1.043

Intraparticle diffusion model (IDM)							
[ASA] (mg L^–1^ **)**	*C* (mg g^–1^)	*k*_id_ (mg g^–1^ min–1/2)	*R* ^2^	*R* ^2^_adj_	SSE	ARE (%)	RMSE
1	1.301 ± 0.065	0.169 ± 0.008	0.371	0.266	0.058	0.914	0.242
5	3.945 ± 0.197	0.651 ± 0.033	0.391	0.290	6 × 10^–22^	2 × 10^–11^	2 × 10^–11^
10	1.351 ± 0.068	1.840 ± 0.092	0.893	0.875	0.066	0.268	0.258
30	6.239 ± 0.312	3.406 ± 0.170	0.596	0.528	5 × 10^–15^	2 × 10^–8^	7 × 10^–8^
50	9.621 ± 0.481	5.414 ± 0.271	0.688	0.636	6 × 10^–22^	4 × 10^–12^	2 × 10^–11^

**Figure 10:**
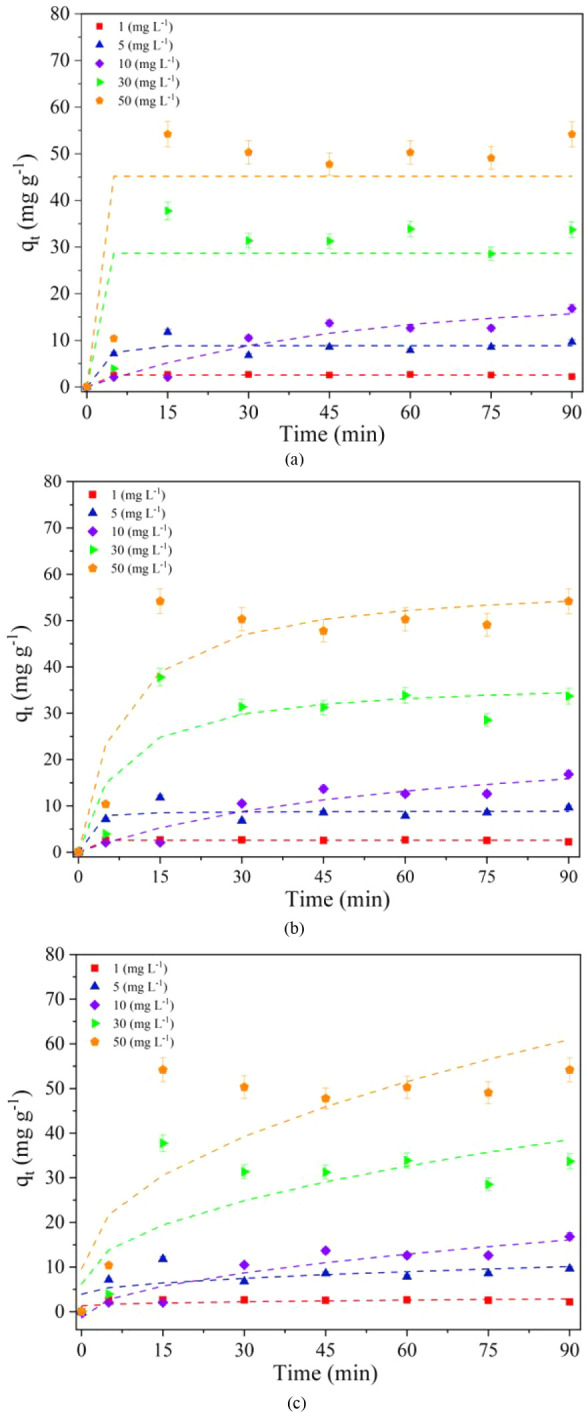
Kinetic curves for the ASA drug onto biochar of (a) PFO, (b) PSO, and (c) IDM (biochar concentration = 0.25 g L^–1^ and pH 4).

As we can see in Table 5, the kinetic evaluation revealed that, at low ASA concentrations (1–10 mg L^–1^), the pseudo first-order (PFO) model presented the best fit (*R* ^2^ > 0.81), indicating a predominance of fast adsorption at surface sites and physical interactions. At higher concentrations (30–50 mg L^–1^), the pseudo second-order (PSO) model was more adequate (*R* ^2^ up to 0.864), suggesting an increasing contribution of specific chemical interactions at higher-energy sites. The intraparticle diffusion model (IDM), although presenting good correlation coefficients (*R* ^2^ > 0.89), did not explain the process alone, indicating that internal diffusion acts in conjunction with surface phenomena. Thus, ASA adsorption follows a multiphase mechanism, dependent on the concentration and availability of active sites. The PSO model is based on the premise that the adsorption rate is proportional to the square of the amount of adsorbate still available, implying a stronger dependence on specific interactions and the limited availability of active sites.^77^ Figure 10 shows the plot of the studied kinetic models: (a) PFO, (b) PSO, and (c) IDM.

### 3.5 Adsorption Thermodynamics

Table 6 shows the values obtained in the thermodynamic study. Δ*S* ^0^, when positive, indicates an increase in the entropy of the process. A positive Δ*H* indicates an endothermic process, thus absorbing heat. Furthermore, the magnitude of Δ*H* ^0^ indicates how the adsorbent interacts with the adsorbate. This interaction occurred by physisorption through van der Waals forces.^78^ Typically, Δ*H* ^0^ values are less than 20 (kJ mol^–1^), which was previously confirmed in the kinetic study at the same ASA concentration, and Δ*G* ^0^, which has negative energy, indicates that the process occurs spontaneously.

**Table 6:** Thermodynamic Parameters for the Adsorption of ASA Drug onto Biochar

*T* (K)	*K*_c_ (L g^–1^ **)**	Δ*G* ^0^ (kJ mol^–1^)	Δ*H* ^0^ (kJ mol^–1^)	Δ*S* ^0^ (kJ mol^–1^ K^–1^)
298.15	2.08	–1.8	27.38	0.09
308.15	3.14	–2.8		
318.15	4.16	–3.8		

Operational conditions: [ASA] = 10 mg L^–1^ | [Biochar] = 0.25 g L^–1^ | pH = 4|.

### 3.6 Adsorption Mechanism

It was possible to identify the type of adsorption that occurs in the process, identifying the best equilibrium model, which was the Khan isotherm. This model indicates a combination of both Langmuir and Freundlich, suggesting that adsorption can occur in a hybrid form as a monolayer and multilayer.^80^ In kinetics, lower concentrations (1, 5, and 10 mg L^–1^) had a better fit for PFO, suggesting physical adsorption, while higher values (30 and 50 mg L^–1^) had a better fit for PSO, suggesting chemical adsorption.^81^ Finally, with the thermodynamic study, Δ*H* ^0^ was lower than 20 kJ mol^–1^, indicating adsorption with weak forces (van der Waals) that is physical.^82,83^ Therefore, the adsorption occurred predominantly in a physical way by electrostatic action, interacting with the surface of the biochar that adsorbed the ASA in a multilayer and monolayer hybrid, according to Figure 11. Table 7 shows that adsorption mechanisms vary according to the modification method and the type of pollutant. In the present study, gourd biochar treated with H_3_PO_4_ exhibited ASA removal predominantly through electrostatic interactions, which was attributed to the presence of oxygenated functional groups capable of modulating the surface charge. In contrast, other studies have reported mechanisms based on π–π electron donor–acceptor (EDA) interaction and hydrophobic effects (atrazine and methylene blue), associated with the high aromaticity of biochar at high temperatures, or even on hydrogen bonds (malachite green and amoxicillin), favored by polar groups of the adsorbates. These results highlight that acid modification directs adsorption toward electrostatic processes, distinguishing it from other reported mechanisms and reinforcing selectivity against ionizable pollutants.

**Figure 11:**
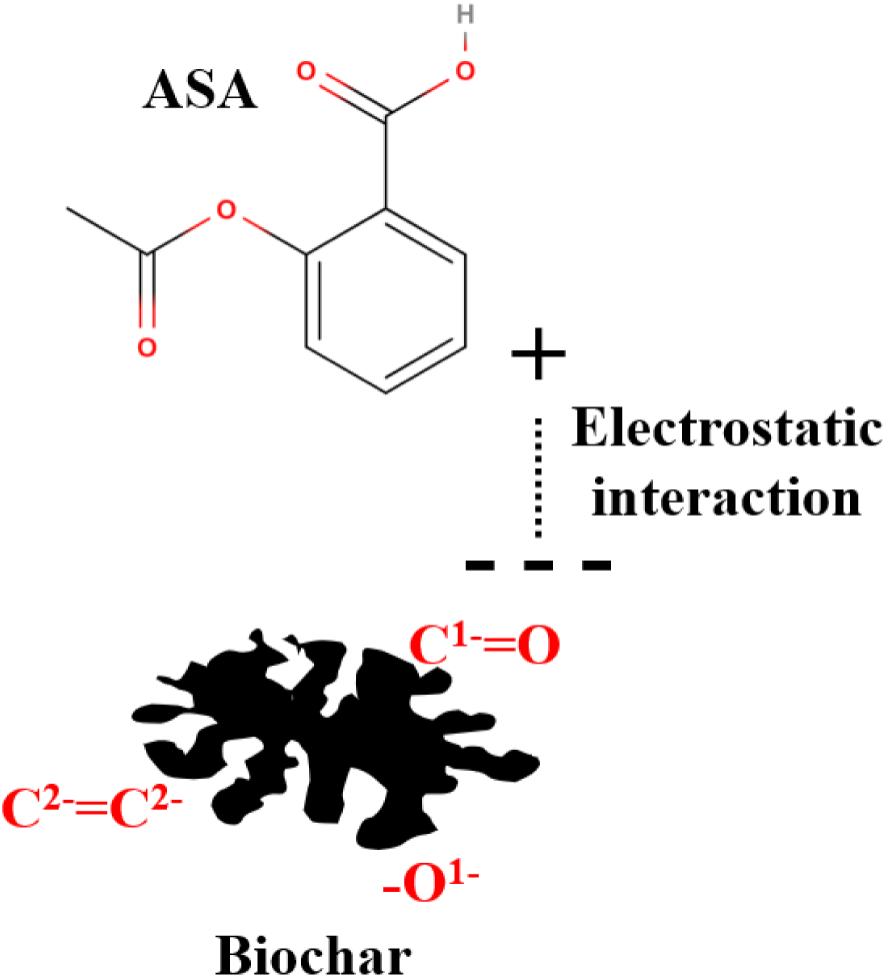
ASA adsorption mechanism using biochar from *Lagenaria siceraria* waste.

**Table 7:** Adsorption Capacities of Biochar for ASA and Other Organic Pollutants

Modification method	Biochar feedstock	Temperature (°C)	Pollutant	*q*_max_ (mg g^–1^)	Removal mechanism
Acid bath (H_3_PO_4_)	Sawdust gourd	600	ASA	137	Electrostatic interaction
N doping (KOH NaN3)^84^	Corn stalks	700	Atrazine	104	π-π EDA Hydrophobic effect
Biological modification^85^ (*Arthrobacter sp*. ZXY-2 *Aspergillus niger* Y3)	Maize straw	700	Atrazine	103	π-π EDA Hydrophobic effect
Acid bath Microwave (H_3_PO_4_)^86^	Plantain Leaf	110	Malachite green	266	π-π EDA H-bonding Electrostatic interaction
Pyrolysis with N_2_ ^87^	Rice straw	500	Methylene blue	50	π-π EDA Electrostatic interaction
Corn cob (ZnCl_2_)^88^	Corn cob	700	Amoxicillin	175	n-π interactions hydrogen bonding

### 3.7 Ecotoxicity Tests

Figure 12 shows the results obtained from the ecotoxicity tests, where ASA exhibited signs of toxicity at a concentration of 200 mg L^–1^. Shortly thereafter, the lethal dose 50% (L_D50_) was approximately 264 mg L^–1^, concentrations above 300 mg L^–1^ resulted in a lethal dose of 100% (L_D100_). For the biochar toxicity tests, within the range of 100–1000 mg L^–1^, the adsorbent demonstrated no toxicity to *Artemia salina*.

**Figure 12:**
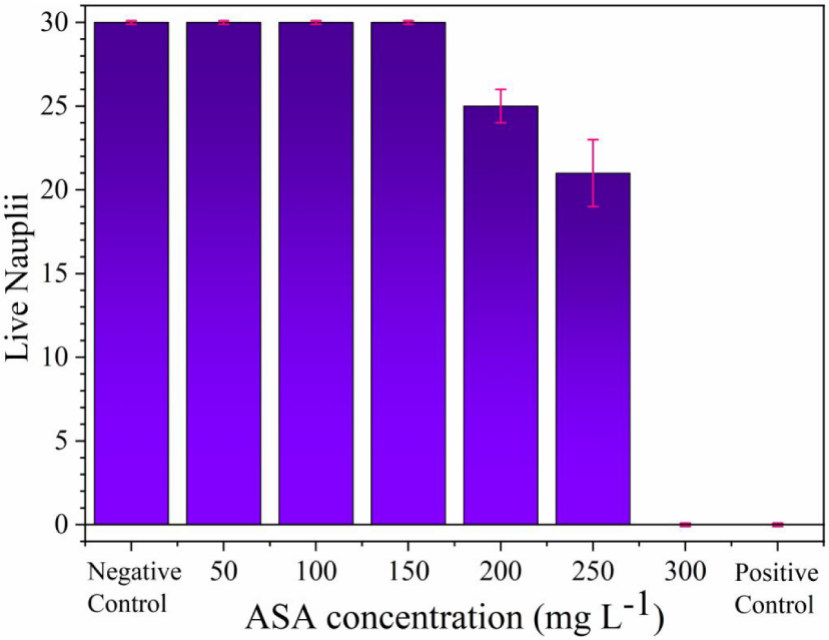
Ecotoxicity tests performed with *Artemia salina* .

Table 8 shows the acute (48 h) toxicity results of ASA for different test organisms, revealing a significant difference in sensitivity between species. The freshwater cladoceran *Ceriodaphnia silvestrii* was the most sensitive, presenting an LD_50_ value of 69 mg L^–1^, followed by *Daphnia sp*. with 88 mg L^–1^. For *Daphnia magna*, LD_50_ values ranged from 136 to 310 mg L^–1^, indicating possible variations between strains or experimental conditions. The marine organisms *Artemia salina* (264 mg L^–1^) and *Artemia* sp. (305 mg L^–1^) showed the lowest sensitivities, suggesting greater resistance to the drug compared to that of cladocerans. These data support the idea that ASA toxicity varies by species and that freshwater organisms, especially of the genus *Ceriodaphnia*, may be more vulnerable to this emerging contaminant in aquatic environments.

**Table 8:** Assessment of Acute Toxicity of ASA after 48 h in Different Organisms

Organisms	LD_50_ (mg L^**–**1^)	Reference
*Daphnia magna*	310	^89^
*Ceriodaphnia silvestrii*	69	^90^
*Daphnia*	88	^91^
*Daphnia magna*	136	^92^
*Artemia sp*.	305	^93^
*Artemia Salina*	264	In this study

## 4 Conclusion

In the present study, it was possible to develop and characterize a novel biochar from *Lagenaria siceraria* waste, as well as to determine the ideal adsorption process conditions for ASA removal (10 mg L^–1^ of ASA, 0.25 g L^–1^ of biochar, and pH 4). The adsorption isotherm data showed an excellent fit with the Khan isotherm model (*R* ^2^ = 0.999), which indicates a hybrid adsorption mechanism involving both monolayer and multilayer adsorption. Kinetic analysis revealed that the PFO model provided the best fit (*R* ^2^ = 0.974) under most conditions, indicating that the adsorption process is predominantly controlled by physical interactions, such as van der Waals forces and electrostatic attractions. The enthalpy change ΔH^0^ values were consistently below 20 kJ mol^–1^, characteristic of physical adsorption mechanisms, while the negative ΔG values confirmed the spontaneity of the adsorption process. Ecotoxicity tests using *Artemia salina* revealed that ASA exhibited a lethal dose (LD50) at 300 mg L^–1^, indicating moderate toxicity in aquatic environments. In contrast, biochar showed no measurable toxicity within the tested concentration range of 100–1000 mg L^–1^. Therefore, the novel biochar demonstrated excellent performance for ASA removal under various conditions, showing strong adsorption capacity, temperature resilience, and environmental safety. These results position biochar as a sustainable and cost-effective alternative for the remediation of organic pollutants in water, with potential applications in wastewater treatment systems and environmental protection initiatives.

## Supplementary Material



## Data Availability

The data underlying this study are available in the published article and its online Supporting Information.

## Notes

Additional experimental details, materials, and experimental design, including the UV–vis scanning of the ASA drug, the calibration curve of the ASA drug, and CCRD 2^3^ results (PDF)

## Notes

M.L.d.C.: Conceptualization, data curation, formal analysis, investigation, methodology, writing—original draft, writing—review and editing. L.R.O.: Data curation, formal analysis, writing—review and editing. G.P.: Data curation, formal analysis, writing—review and editing. J.A.S.T.: Data curation, formal analysis, writing—review and editing. D.C.R.E.: Data curation, formal analysis, writing—review and editing. Y.P.M.: Data curation, formal analysis, writing—review and editing. D.M.D.: Data curation, formal analysis, writing—review and editing. S.N.L.: Data curation, formal analysis, writing—review and editing. W.L.D.S.: Conceptualization, data curation, formal analysis, investigation, methodology, supervision, validation, writing—original draft, writing—review and editing
