# Supplementary material for: A Novel Biochar from Agro-Industrial Waste: Synthesis, Characterization, and Application for Acetylsalicylic Acid Removal
Source: ACS Omega. 2025 Nov 18;10(47):57532–44. doi: 10.1021/acsomega.5c08131 (PMC12676325; doi:10.1021/acsomega.5c08131)
Supplement: Supplementary file 1 [file ao5c08131_si_001.pdf]

Supplementary Information (SI)

A novel biochar from agro-industrial waste: Synthesis, characterization and application for acetylsalicylic acid removal

Matheus Londero da Costa[1], Leandro Rodrigues Oviedo[1], Giovani Pavoski[2], Jorge Alberto Soares Tenório[2], Denise Crocce Romano Espinosa[2], Yolice Patricia Moreno[3,4], Daniel Moro Druzian[1], Sthéfany Nunes Loureiro[1], William Leonardo da Silva[1*]

[1]Applied Nanomaterials Research Group (GPNAp)

Franciscan University (UFN), Santa Maria-RS, Brazil

[2]Polytechnical School of Chemical Engineering

University of the Sao Paulo (USP), São Paulo-SP, Brazil

[3]Strategic Technologies Center of Northeast (CETENE), Recife-PE, Brazil

[4]Department of Fundamental Chemistry (DQF)

Federal University of Pernambuco (UFPE), Recife – PE, Brazil

S.1 Material and Methods

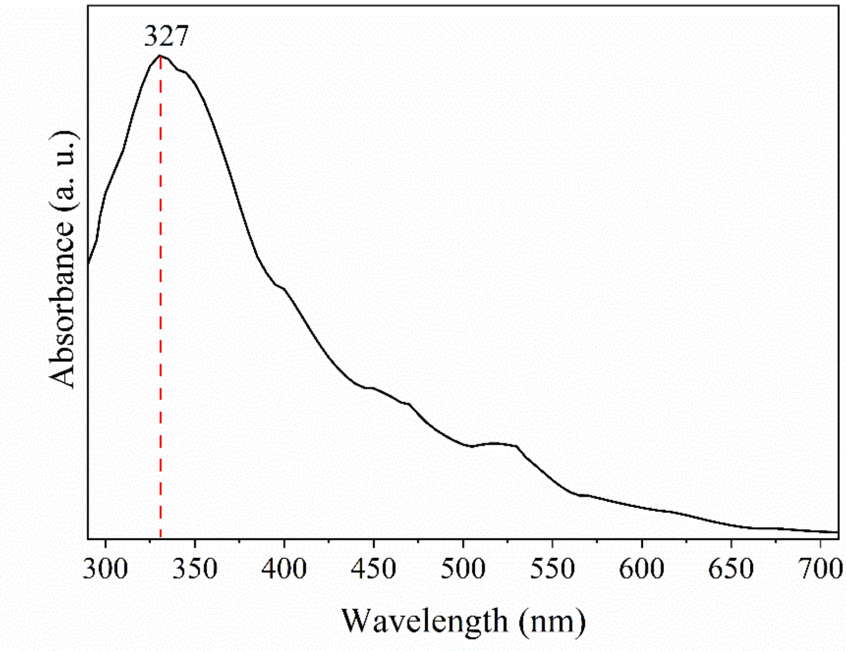

Figure S1: Scanning of ASA in the spectrophotometer.

* Corresponding author.
E-mail address: Williamleonardo_silva@hotmail.com (W.L. da Silva).

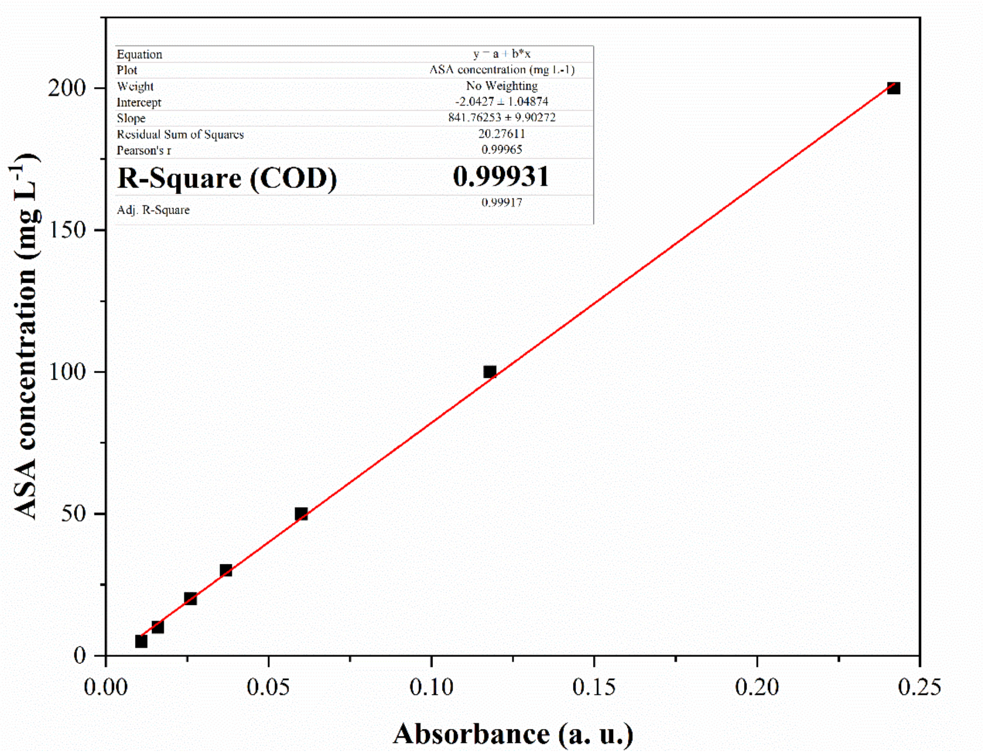

Figure S2: Calibration curve of the ASA drug.

Table S1: CCRD 2^3 results for removal of the drug ASA.

[ASA] (mg L^{-1})	[Biochar] (g L^{-1})	pH	Removal (%)
10.00	0.75	4.00	0.10
30.00	0.75	10.00	0.10
30.00	0.75	4.00	11.6
3.27	0.50	7.00	62.0
20.00	0.50	7.00	34.6
30.00	0.25	4.00	31.5
20.00	0.50	12.02	4.9
30.00	0.25	10.00	20.0
20.00	0.50	1.98	24.3
20.00	0.50	7.00	36.0
20.00	0.50	7.00	37.1
10.00	0.25	4.00	42.0
20.00	0.92	7.00	7.4
20.00	0.08	7.00	29.8
36.73	0.50	7.00	4.4
10.00	0.75	10.00	0.10
10.00	0.25	10.00	22.8
10.00	0.75	4.00	0.10
